# Large-Scale, Fine-Grained, Spatial, and Temporal Analysis, and Prediction of Mobile Phone Users’ Distributions Based upon a Convolution Long Short-Term Model

**DOI:** 10.3390/s19092156

**Published:** 2019-05-09

**Authors:** Guangyuan Zhang, Xiaoping Rui, Stefan Poslad, Xianfeng Song, Yonglei Fan, Zixiang Ma

**Affiliations:** 1 College of Resources and Environment, University of Chinese Academy of Sciences, Beijing 100049, China, zhangguangyuan16@mails.ucas.ac.cn (G.Z.); xfsong@ucas.ac.cn (X.S.); fanyonglei17@mails.ucas.ac.cn (Y.F.); 2 School of Earth Sciences and Engineering; Hohai University; Nanjing 211000, China; 3 Queen Mary University of London, London E1 4NS, UK; stefan.poslad@qmul.ac.uk (S.P.); zixiang.ma@qmul.ac.uk (Z.M.)

**Keywords:** population density, deep learning, mobile phone data, spatial–temporal data analysis and prediction, kernel density estimation (KDE), long short-term memory (LSTM), convolution LSTM (ConvLSTM), autoregressive moving average (ARMA)

## Abstract

Accurate and timely estimations of large-scale population distributions are a valuable input for social geography and economic research and for policy-making. The most popular large-scale method to calculate such estimations uses mobile phone data. We propose a novel method, firstly based upon using a kernel density estimation (KDE) to estimate dynamic mobile phone users’ distributions at a two-hourly scale temporal resolution. Secondly, a convolutional long short-term memory (ConvLSTM) model was used in our study to predict mobile phone users’ spatial and temporal distributions for the first time at such a fine-grained temporal resolution. The evaluation results show that the predicted people’s mobility derived from the mobile phone users’ density correlates much better with the actual density, both temporally and spatially, as compared to traditional methods such as time-series prediction, autoregressive moving average model (ARMA), and LSTM.

## 1. Introduction

Mapping population dynamics is of great significance for city and transport planning [1,2,3], public safety warning [4,5,6], disaster impact assessments [7,8,9], and epidemic modeling [10,11,12]. The analysis of mobile phone data is frequently used to map the spatial and temporal situation of users [13,14]. However, a more fine-grained temporal resolution of dynamic population distributions is still a challenge when studying human activities [13]. There is a range of motivations and applications for doing this. For instance, the prediction of people density could help drive improved regional authority policy decisions, so as to help avoid stampedes such as the tragic Shanghai Stampede, when 36 people died and 49 were injured during the New Year’s Eve celebration on 31 December 2014 in the Shanghai Bund [6]. Another example is that there are a large number of shared bicycles in many big cities, especially in China, where bicycles could be better distributed according to the dynamic movement of the population in order to increase revenue [15]. Takeaway-food delivery could be made more efficient if service providers were aware in advance where and when an area holds a strong potential purchasing power [16].

There is a range of sensors that could be used to profile users’ mobility, especially in urban areas. For example, fixed sensors at traffic lights can be used to predict when road congestion at those locations will occur and disappear [17]. Sensors such as global positioning system (GPS) can be used, either fixed in vehicles [18] or in mobile phones that can accompany users as they move about [19]. However, these mobile sensing techniques face several challenges. Firstly, the sensor data have to be acquired via a mobile app and uploaded, and there are no such apps that have good coverage and that freely share their mobility data. There are privacy concerns even if the mobility data are anonymized [20]. The use of mobility sensors and associated apps can be energy-hungry such that a device running such an app can more quickly run out of energy and shut down [21]. Our method to collect data from the mobile phone network overcomes each of these challenges provided that a mobile phone network operator shares the data. Its key characteristics are that (1) many people have mobile phones; (2) only the time and location of the phone within a cell of the network is recorded (thus, we cannot derive the exact address of someone’s home location); (3) the method is low-energy because it just relies on a mobile phone cell’s wireless access point to periodically send “ping” signals to check if a mobile phone receiver is within its range.

Mobile phone networks, also called cellular networks, are composed of cells, i.e., geographic zones around a phone tower or wireless network access point. Each mobile phone can be located by identifying the geographic coordinates of the transmitting tower it currently communicates with, within its associated cell. Furthermore, when a user makes a call or sends a text message, these will be recorded in the call detail record (CDR) which is located at its nearest phone tower. CDRs are not as structured as traditional travel survey data, which contain location and time information for meaningful activity destinations, or as precise as global navigation satellite system (GNSS) data, which provide location data with higher frequency and accuracy [22]. However, as a by-product for billing purposes carried out routinely by mobile service carriers, CDR data can be obtained at a much lower cost and on a greater scale than GNSS data [23]. Reference [14] highlights how facilitating access to anonymized mobile phone data might enable fast and cheap production of population maps in emergency situations and in data-scarce environments, e.g., in rural or less accessible regions where there is less digital infrastructure such as public transport, local area networks, etc.

Although, the global mobile phone penetration rate (i.e., the percentage of active mobile phone subscriptions within the population) reached 100% in developed countries in 2014. In contrast, in developing countries, it is about 90%; however, this continues to rise [24]. Note that mobile phone density may not be equal to population density because of periods when the phones are not in use such as at nighttime or during daytime because one person may have more than one phone, more than one subscription with the same phone, or have no phone. Deville et al. [14] used active users and active records of mobile phones to estimate the population and to characterize the relationship between users’ phone use density and the actual population density. This was used in several applications [25,26,27,28]. However, using census data which can be collected by systematically acquiring and recording information about the members of a given population is often not very accurate to study a dynamic population distribution [13].

Some studies (see Section 2) separate daytime, e.g., from 7:00 a.m. to 8:00 p.m. and nighttime as 8:00 p.m. to 7:00 a.m. and then use cell mobile metadata to calculate the users’ density at night. Some other studies use the census data, which are infrequently collected by the government, i.e., every 10 years, to estimate the census population density and the actual population density. Therefore, in this study, we use the mobile cell phone data which can directly reflect the phone users’ distribution to characterize people’s mobility based on the ubiquitous nature of mobile phones in modern society [14]. As machine learning and deep learning techniques are currently popular to predict or estimate output values in many fields [29], in this study, a deep learning approach is also proposed for estimating and predicting mobile users’ dynamic distribution that potentially contributes to mapping and predicting a population’s dynamics at a more fine-grained spatial scale and temporal scale, i.e., every two hours, compared to two traditional methods which can be used as a baseline, i.e., the autoregressive moving average (ARMA) model and a popular deep learning method that uses a long short-term memory (LSTM) model for predicting time-series data.

The remainder of this article is organized as follows: Section 2 reviews related work. Section 3 presents the mobile phone dataset from 2 February 2015 to 17 February 2015 in Beijing. Section 4 presents the methods for calculating users’ distributions and predicting their density using the ARMA model, LSTM model, and convolution LSTM (ConvLSTM) model. In Section 5, we evaluate their accuracy and compare these three methods’ abilities to predict the urban population distribution both in time and space. In Section 6, we present our conclusions and our thoughts for future research.

## 2. Related Work

The focus here is on mapping mobile phone users’ distribution which can present the mobility of people and contribute to the estimation of the real dynamic population distribution in space and time. Furthermore, the focus is also on predicting mobile phone users’ spatial and temporal distribution in the future, forecasting, at least one whole day in advance, with high accuracy.

In terms of population mapping, most previous studies concentrated on using simple areal-weighting methods (a technique for estimating the values of overlapping but incongruent polygon features) [29,30,31,32] and estimating the population data from the census data with respect to the census administrator units or regions [33]. Others used some ancillary data such as remote-sensing images, land-use data, e.g., urban or sub-urban boundaries, to estimate population counts within the census units. However, the census and ancillary data used in these previous studies usually lag behind the time of interest significantly because a census enumeration is recommended about only once every ten years by the United Nations (UN) [34]. It is really is a challenge to acquire population data for such a large urban area at a daily or even higher temporal resolution [13]. In recent years, advances in information communication technology (ICT) and the increased accessibility of location-aware mobile devices provided multi-source location-based big data about human activity [35]. Combined with other ancillary data, mobile data were utilized in modeling human mobility [36,37,38] and in making dynamic estimates of population distribution more feasible [13,14]. Furthermore, some efforts on nowcasting, i.e., forecasting changes in the near future of the population distribution, using mobile phone data, were undertaken; for example, Reference [39] used CDRs and a machine learning approach to estimate residents’ distribution within a territory. Reference [40] proposed an analytical framework based on CDRs to nowcast the population count, well-being, and economic development. Note that there is no standard definition of what near future means. Because of the physical inertia to make detectable changes in the physical environment, nowcast takes the order of hours, rather than minutes, within this context. In some cases, it is not clear how far in the future the estimate is made for (e.g., see References [39,40]).

An autoregressive moving average model (ARMA) is used traditionally to predict future values of a time series in several important fields of interest such as linear prediction, system identification, and spectral analysis [41,42,43]. More recently, some studies and applications focused on the use of machine learning and deep learning, especially for spatial prediction such as traffic flow prediction [17,44,45,46,47], precipitation prediction [48,49,50], and some prediction of social issues [51,52].

Among the deep learning models, Long Short-Term Memory (LSTM) achieved a good performance within the field of natural language (NL) processing [53] and trajectory prediction [54]. Although the accuracy of the LSTM model is high, its input data are one-dimensional, and it is not suitable for spatial sequence data such as video, satellite, or radar image datasets; hence, the LSTM model is often combined with other models. A CNN–LSTM algorithm combines a convolutional neural network (CNN) with LSTM such that the CNN part of the model processes the data and the one-dimensional result then feeds into an LSTM model. For example, a CNN–LSTM algorithm was proposed and applied by Liu et al. [55] and Rad et al. [56]. The former’s experimental results on the MNIST and FashionMNIST datasets show that the algorithm is universal. For example, it was used for image recognition and for classification tasks. The experiments for the latter show that transferring the raw feature space to a dynamic feature space via the proposed architecture enhances the performance of an automatic stereotypical motor movement (SMM) detection system, especially for skewed training data when using a CNN–LSTM, to model the temporal patterns in the sequence of multi-axis Inertial Measurement Units (IMUs) signals in terms to the spatial and temporal data. Although related to CNN–LSTM, a convolution LSTM (ConvLSTM) network is different in that ConvLSTM replaces the LSTM matrix multiplication with a convolution operation at each gate in the LSTM cell. For example, ConvLSTM was proposed to build an end-to-end trainable model for rain or precipitation when capturing spatiotemporal correlations [50]. Liu et al. used a ConvLSTM module to analyze historical traffic flow data and to validate that it can achieve better prediction accuracy compared with existing approaches [57]. Qiao et al. proposed a time-distributed ConvLSTM model to extract spatiotemporal features of multi-sensor time series for health monitoring [58]. Yuan et al. proposed a Hetero-ConvLSTM framework, where they incorporated spatial graph features and a spatial model ensemble to address the spatial heterogeneity of the data, e.g., in urban versus rural regions. The extensive experiments show that the proposed framework makes reasonably accurate predictions and significantly improves the prediction accuracy over baseline approaches [59].

In conclusion, spatial–temporal population estimates of mobile phone users extracted from CDRs may exhibit unstable time sequences across some neighboring cells. ARMA is a simpler and more efficient model which is suitable for a time series with stationary values that vary at a relatively small scale; however, this suffers from poor accuracy for population estimates. As a popular deep learning model, LSTM is currently being used for a wide range of predictions for time-series data with good accuracy, but it does not take into account the effects of dissimilar neighboring spatial features, which can make the time prediction less accurate. ConvLSTM improves LSTM in that its use of convolutions can improve the impact of spatial autocorrelation (see Section 4).

## 3. Data

**Figure 1:**
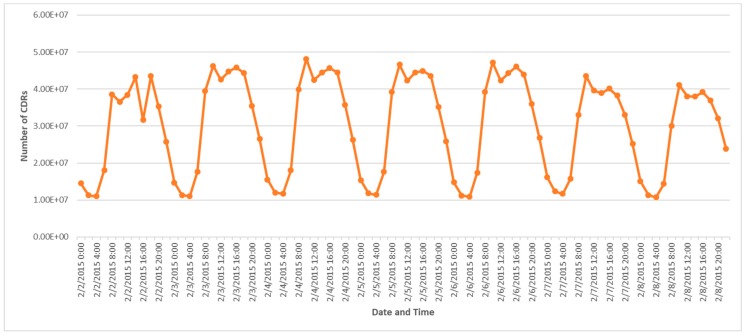
The number of call detail records (CDRs) distributed every 2 h over one week.

In this study, we used the anonymized individual call detail records (CDRs) that contain information about user identifiers (IDs), time, and corresponding base station locations (Table 1). The dataset was collected anonymously for scientific research. Whenever a user calls or sends a text message provided by his or her Chinese mobile phone operator, a new data record is recorded. All personally identifiable information is masked. The dataset for this study includes over 4.8 billion records of more than 300 million users per day from 2 February to 17 February 2015 in Beijing, China. Users are more active during the daytime than at night both on weekdays and weekends (Figure 1).

We used the locations of all 51,216 mobile base stations based upon their unique index (Table 2). The coverage area of each mobile base station can be approximated as the Voronoi polygon that surrounds it. When a phone is used to make a call or send a text message, its location is found through verifying the range of the specific mobile base station to which the phone is connected.

## 4. Method

**Figure 2:**
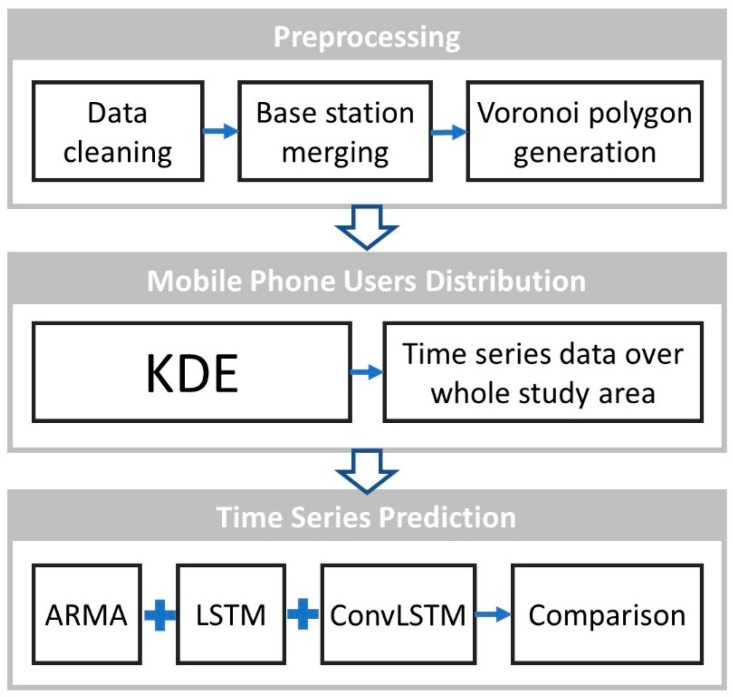
Method overview to perform a temporal and spatial prediction of mobile phone users.

Our method overview is as follows: we firstly extracted the CDRs of mobile users every two hours (this time window was selected as a tradeoff between the higher computation cost needed for a shorter time window and the greater spatial variance of CDRs if a longer time window is used). In addition, only the first 30 s of CDRs for each 2-h time window per base station was analyzed as being a representative sample of user mobility in order to further reduce the data to make it more manageable for data analysis, yielding 160 MB of data (for 30 s) instead of 42 GB (for 2 h). There are two justifications for analysis of 30-s data segments every 2 h: (1) to reduce the data analysis to make computation more accessible in poorer world regions where they may lack a more costly data processing infrastructure to perform such an analysis; (2) most users use their smart phone for less than 30 s [60]; hence, 30 s of CDR encompasses the start and end of most calls and texting. The user density within the whole study region was generated from a kernel density analysis, sequencing the period of 16 days and structuring this as a raster image that consisted of a matrix of cells (or pixels) organized into rows and columns (or a grid), where each cell contained a value representing the user density as Voronoi polygons. Finally, a traditional ARMA model, a deep learning long short-term memory (LSTM) model, and an LSTM model combined with convolution were used to train and predict the temporal and spatial density distribution of mobile phone users, and their accuracy was calculated and compared. An overview of the method to determine the temporal spatial distribution of users is given in Figure 2.

### 4.1. Data Prepocessing

**Figure 3:**
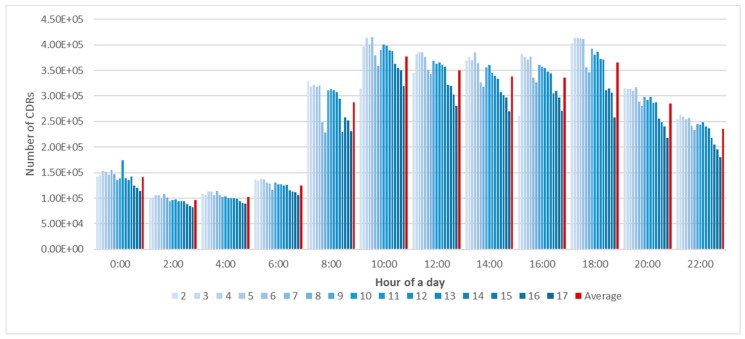
Variation in the number of CDRs for the first 30 s every 2 h over 16 days (2–17 February).

Firstly, we cleaned the data using three steps as follows: (1) to simplify the amount of data analysis, we considered 2-h time windows for analysis starting from 12:00 a.m. during each of the 16 days in the dataset, generating 192 time-series data files or records; (2) for every 2 h, the first 30 s of data were analyzed as a representative sample for each record phone call or text message, with each user being regarded as being stationary within a Voronoi polygon region (Figure 3); (3) there were a few no-value data fields for some records such as latitude and longitude (which could, for example, have been caused by power outages and data loss during data communication). These were regarded as outliers and deleted.

Secondly, because the accuracy of the latitude and longitude of mobile phone base stations is not enough when some base stations are located on top of buildings or otherwise close to each other (this varies depending on the phone cell range), we combined such base stations which had the same latitude and longitude. Hence, after combining 17,447 base stations, we matched the records to the corresponding base stations over the whole time sequence.

**Figure 4:**
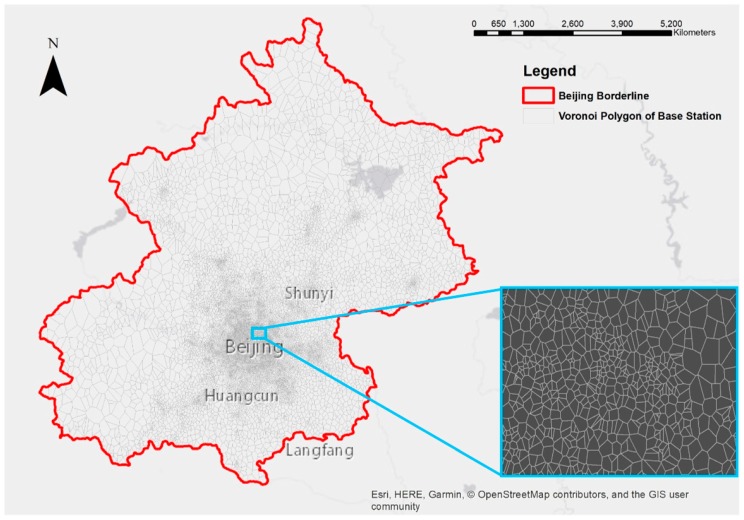
Mobile base stations are represented by Voronoi polygons in Beijing.

Finally, a Thiessen polygon algorithm [61] was utilized to create a Voronoi polygon for each mobile phone base station in order to define the location of phone users within a district (Figure 4). Then, for each polygon corresponding to each base station, we generated random points to represent the distribution of phone users as one point per user.

### 4.2. Modeling Mobile Users’ Population Distribution Using Kernel Density Estimation (KDE)

We cannot simply input the locations of phone users as the counts in each Voronoi polygon into the neural network to do a time-series data prediction, e.g., using ConvLSTM (see Section 4.3), because they are impossible to input into ConvLSTM because of their irregular shape. Instead, we firstly performed a nonparametric estimation of the distribution of mobile phones to convert them into a set of counts for regular resized grid cells. To calculate this distribution, an appropriate search radius was calculated by geographic information system (GIS) software, e.g., ArcGIS (Available from http://desktop.arcgis.com/en/arcmap/, retrieved 01/11/2018), to produce an estimation of the density at different spatial resolutions, such as 800 m, before producing a raster grid consisting of square 800 m × 800 m cells for input into ConvLSTM. This could also be transformed to other spatial resolutions, such as a grid of square cells 5 km in size in order to decrease the calculation cost of the model allowing it to be performed on a regular personal computer (PC) without any special hardware acceleration such as a graphics processing unit (GPU).

Kernel density estimation (KDE) was used here to perform an automatic search to explore the hotspots of the event distribution, and this method uses complex distance attenuation to measure changes in event density [62,63,64]. A GIS-based KDE estimation method mainly uses a moving window to calculate and output the point or line density of each grid cell. Given the sample $\left(x_{1},x_{2},\ldots ,x_{n}\right)$ is an independent identically distributed (iid) sample extracted from the population with a distribution density function f at a point x, f(x), calculated using a Rosenblatt–Parzen kernel estimate, we get the following:

(1) $$f_{n}\left(x\right)=\frac{1}{nh}\overset{n}{\underset{i=1}{\sum }}k\left(\frac{x-x_{i}}{h}\right),$$

where k is the kernel function, h > 0 is a smoothing parameter for the kernel called the bandwidth, and $\left(x-x_{i}\right)$ is the distance from the estimated point *x* to a sample point $x_{i}$.

When KDE is processed, the determination or selection of the bandwidth h has great influence on the calculation result. As h increases, the change of the point density in space is smoother, but the structure of the density is masked. When h is reduced, the estimated dot density change can change very abruptly between Voronoi polygons. In specific applications, it is necessary to test different h values according to the different land use in physical environments, e.g., presence of banks or train stations, in order to explore the nuclear density surface that can match the actual situation.

**Figure 5:**
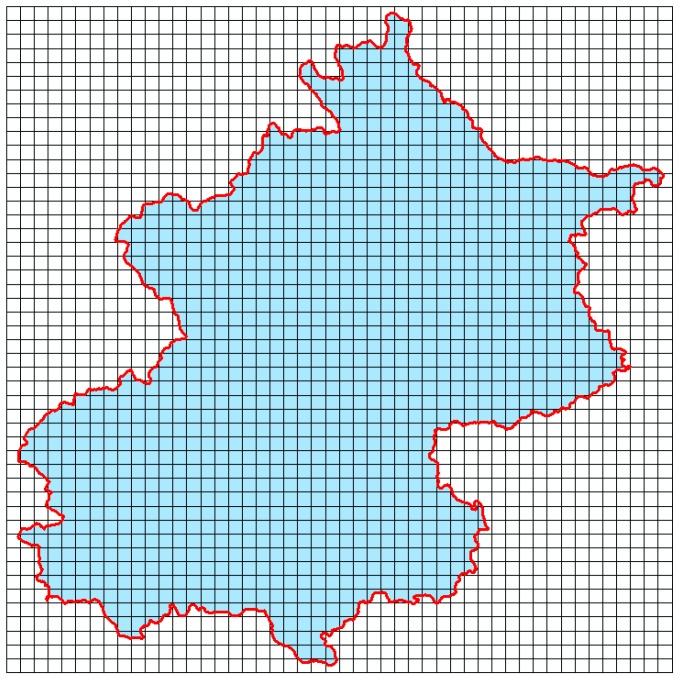
Training grids for the three population density prediction methods: autoregressive moving average (ARMA), long short-term memory (LSTM), and convolution LSTM (ConvLSTM).

The specific steps for KDE estimation are as follows: (1) define a search radius to count the number of events that fall within the circle using a sliding circle; (2) determine the output raster size based on the density accuracy requirements; (3) calculate the density contribution of each event to each grid in the circular domain using the kernel function; (4) assign the density value of each raster to the value of the density contribution of each event in the raster search radius; (5) output the density values for each raster. In this study, we compared the different bandwidths (Figure 5) and finally determined the characteristics of mobile phone users’ distribution for the Beijing area within a 150-km default radius, using the ArcGIS10.5 kernel density estimation tool (See http://desktop.arcgis.com/en/arcmap/10.5/tools/spatial-analyst-toolbox/how-kernel-density-works.htm).

### 4.3. Prediction Models for Time-Series Data

Having created the time-series user density distributions, we introduce an improved prediction model to forecast future user density distributions based on ConvLSTM and then compare its predictions to two baseline models: ARMA and LSTM. Firstly, the baseline systems are described and then the new method is explained.

#### 4.3.1. ARMA Model

The ARMA model consists of two parts, the autoregressive (AR) part and the moving average (MA) part; it is developed using the following equation:

(2) $$S\left(t\right)=\sum_{i=1}^{p}\alpha_{i}S\left(t-i\right)+\sum_{j=1}^{q}\beta_{j}e\left(t-j\right),$$

where *S*(*t*) is the predicted mobile phone user density at time t. In the AR part, p is the order of the AR process, and $\alpha_{i}$ is the AR coefficient. In the MA part, q is the order of the MA error term, $\beta_{j}$ is the MA coefficient, and e(t) is the white noise that produces random uncorrelated variables with zero mean and constant variance [59]. The future values can be predicted using the realized ARMA model. For example, Equation (3) is applied to predict the hour-ahead forecasting (h = 1, 2, 3, …, hours).

(3) $$S\left(T\right)=\sum_{i=1}^{p}\alpha_{i}S\left(T-i\right)+\sum_{j=1}^{q}\beta_{j}e\left(T-j\right),$$

where S(T)(T=t+h) is the predicted mobile phone user density at time t+h.

In this study, to decrease the amount of computation and usage of the device memory, we separated the whole Beijing area into 48 × 48 rectangle grid cells, in which every cell was a square grid of 5 km × 5 km. Then, the average density value was extracted into every cell to represent the local user density. There was a total of 192 cells generated from the corresponding raster images. Using this method, we separately predicted the values of 12:00 a.m., 2:00 a.m., …, 10:00 p.m. on 17 February 2015 using the history data between 2 February 2015 and 16 February 2015, calculating cells one by one for a total of 2304 times.

#### 4.3.2. LSTM and Convolutional LSTM (ConvLSTM) Models

**Figure 6:**
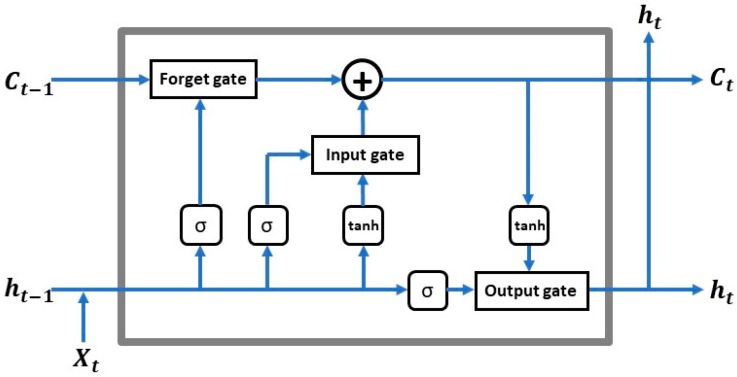
The inner structure of an LSTM cell.

Long short-term memory (LSTM) is a type of recurrent neural network (RNN) node structure known to have good performance when handling time-series data with temporal autocorrelations [59]. It was used to successfully learn and generalize the properties of time sequences such as traffic flow [65] and financial stock option return [66]. The core concept of LSTM is the cell state affected by various interlinked gates. The cell state acts as a transport highway that transfers relative information all the way down the sequence chain as the “memory” of the network. The cell state can carry relevant information throughout the processing of the sequence. Thus, even information from earlier time steps can make its way to later time steps, reducing the effects of short-term memory. As the cell state evolves, information gets added or removed via gates, acting as a type of neural network that decides which information is allowed to exist for the cell state by learning what information is relevant (during training) [67]. In an LSTM network (Figure 6), at each time step t, the hidden state $h_{t}$ is updated by the current data, i.e., at the same time step $X_{t}$, the hidden states at the previous time step $h_{t-1}$, the input gate $i_{t}$, the forget gate $f_{t}$, the output gate ${ο}_{t}$, and a memory cell $C_{t}$ are updated as well [53]. The inner principle of the model is similar to that of ConvLSTM; thus, its equations will not be repeated here, as they are given in the introduction of the ConvLSTM model below.

**Figure 7:**
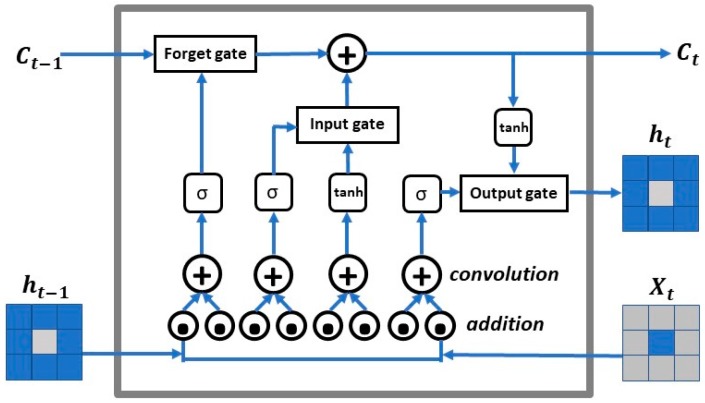
The inner structure of an example ConvLSTM cell.

The ConvLSTM model is a variation of LSTM to handle spatiotemporal prediction problems, which were firstly introduced by Shi et al. [50] for precipitation nowcasting, where nowcasting is a technique for very short-range forecasting of the current state using an estimate of speed and direction of movement. In this paper, we follow the formulation of ConvLSTM as in Reference [50], which includes inputs $X_{1},\ldots ,X_{t}$, cell outputs $C_{1},\ldots ,C_{t}$, hidden states $h_{1},\ldots ,h_{t}$, and gates $i_{t}$, $f_{t}$, ${ο}_{t}$, and uses a three-dimensional (3D) tensor structure. The first two dimensions of the three-dimensional spatial–temporal tensor of each input feature of a ConvLSTM network are the spatial dimensions and the third dimension is time. The input-to-state and state-to-state transitions of the ConvLSTM cell involve convolutional operations that output three-dimensional tensors, as with the original LSTM model (Figure 7). This model can be further formulated using the following equations, where ‘∗’ denotes the convolution operation and ‘∘’ denotes the Hadamard product.

(4) $$i_{t}=\sigma \left(W_{xi}*X_{t}+W_{hi}*h_{t-1}+W_{ci}\circ h_{t-1}+b_{i}\right),$$

(5) $$f_{t}=\sigma \left(W_{xf}*X_{t}+W_{hf}*h_{t-1}+W_{cf}\circ h_{t-1}+b_{f}\right),$$

(6) $${ο}_{t}=\sigma \left(W_{xο}*X_{t}+W_{hο}*h_{t-1}+W_{co}\circ h_{t-1}+b_{ο}\right),$$

(7) $$C_{t}=f_{t}\circ C_{t-1}+i_{t}\circ \tanh\left(W_{xc}*X_{t}+W_{hc}*h_{t-1}+b_{c}\right),$$

(8) $$h_{t}={ο}_{t}\circ \tanh\left(C_{t}\right).$$

In the above equations, $i_{t}$, $f_{t}$, and ${ο}_{t}$ are the outputs of the input gate, forget gate, and output gate for time step t. $C_{t}$ is the cell output at time step t. $h_{t}$ is the hidden state of a cell at time step t. Sigmoid (σ) is used as the gating function for the three gates, since it outputs a value between 0 and 1. It can either let no flow or a complete flow of information through the gates. On the other hand, to overcome the vanishing gradient problem, which is a difficulty found in training artificial neural networks with gradient-based learning methods and backpropagation, a function is needed (tanh) whose second derivative can be sustained for a longer range before going to zero. W and b are weight matrices and bias vector parameters which need to be learned during training. These equations are illustrated in Figure 6 and described above.

ConvLSTM has some useful properties for mobile users’ distribution prediction, as the LSTM part may capture the temporal autocorrelation in the data, and the convolution operator may capture the local spatial features, which are caused by the spatial autocorrelation [68].

We input the same time-series sets as input data into the realized LSTM model for training and prediction. To validate the accuracy of the ConvLSTM model training, a cross-validation method is presented in Section 5.1, explaining how to use ConvLSTM to predict the mobile phone user density. We selected the first 180 combined grids or cells (over the whole 15 days from 2 February to 15 February 2015) to input to the ConvLSTM module for training, and then tested the prediction (17 February 2015) on the last 12 grids to compare its accuracy. This is the same training data versus test data separation that we also used to test the prediction of spatial density for the ARMA and LSTM models.

## 5. Results and Discussion

This section is organized as follows: we firstly show the process and results of the distribution of mobile phone users using the CDRs from a specific telecom network (China Mobile) operator over the period. Then, we test the predictions using the ConvLSTM model and discuss the results and their accuracy using a cross-validation strategy suitable for time-series data. Finally, the predicted results of ARMA, LSTM, and ConvLSTM models are presented, and their accuracies, both at a temporal and spatial scale, are analyzed and compared.

### 5.1. Determination of Mobile Users’ Population Distribution Using KDE

**Figure 8:**
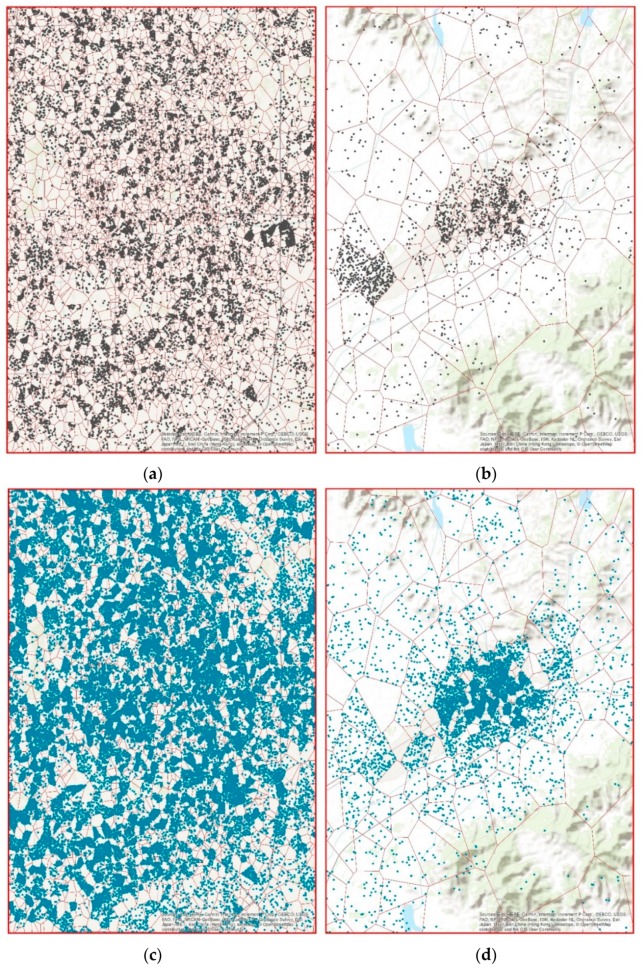

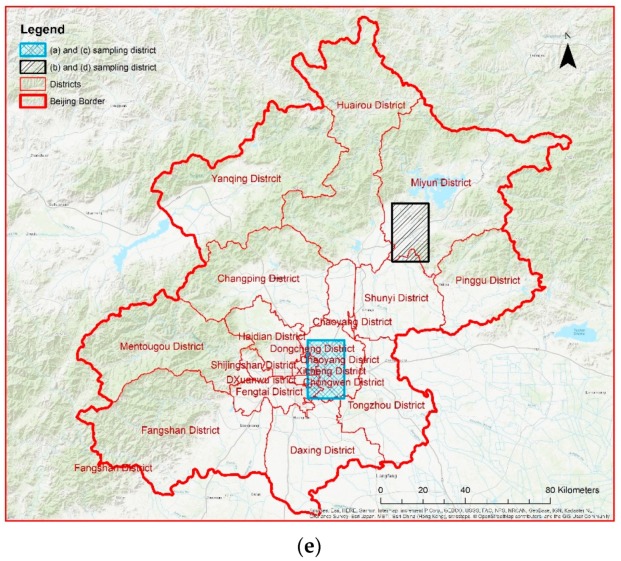
The spatial distribution of a random selection of users within each Voronoi polygon for different areas and times: (**a**) random users within a high-density area at 2:00 a.m.; (**b**) random users within a low-density area at 2:00 a.m.; (**c**) random users within a high-density area at 10:00 a.m.; (**d**) random users within a low-density area at 10:00 a.m.; (**e**) Illustration showing the location of the areas with high and low population density used for the analysis of the time prediction.

Figure 8 gives an example of a random sample of mobile users’ distribution at two time points on 2 February 2015, e.g., 2:00 a.m. and 10:00 a.m., based on the statistic number of records over a 30-s interval. A random sample was used because showing all users would flood the figure making it difficult to see patterns. As Figure 8a shows, at midnight (12:00 a.m.), random users have a relatively high density in core city areas such as the Dongcheng and Chaoyang districts of Beijing, while suburban areas such as the Shunyi and Huairou districts always have a lower density of people using mobile phones, with a lower density of base stations. In contrast, in the daytime (10:00 a.m.), the activity of people using phones increases in both city areas and suburban districts; however, the activity in city areas still shows a much higher level than that in the suburban districts.

**Figure 9:**
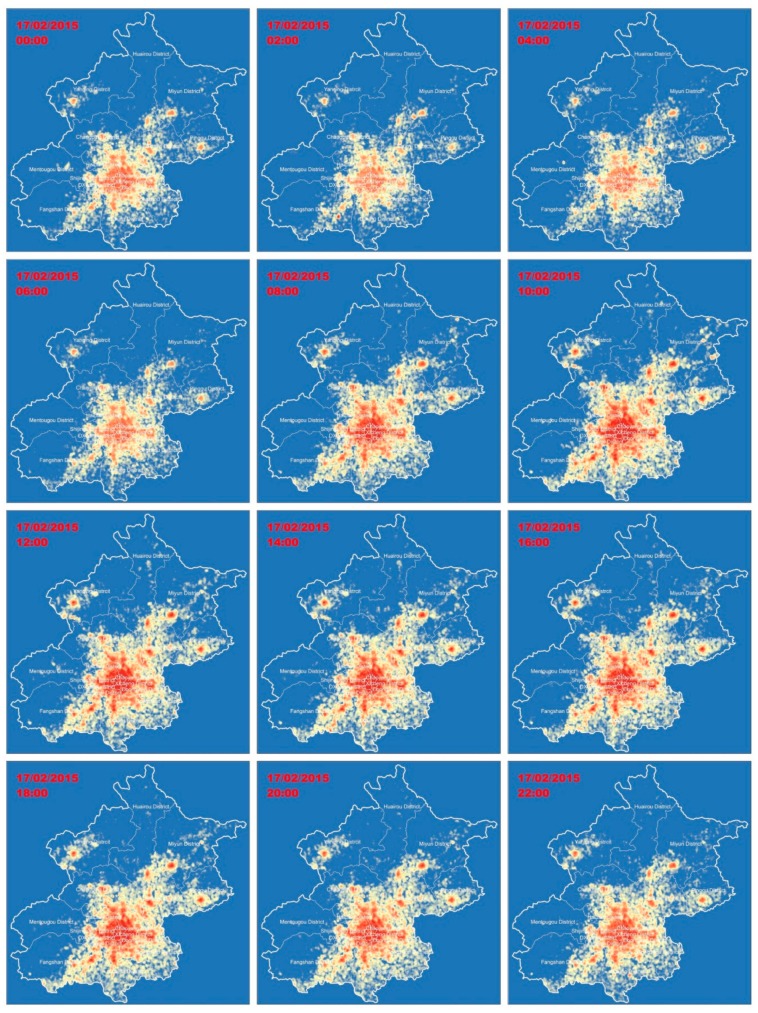
Mobile users’ distribution generated using the kernel density estimation (KDE) method on 17 February 2015 at a higher spatial resolution.

The spatial mobile users’ density distribution on 17 February 2015, the predicted targeted day, was depicted using the kernel density method (see Figure 9). Density was visualized using a geometrical interval to classify this distribution into 15 levels, so as to show the differences in user density in space and time. Mobile phone users mainly gather in the center of the city. For example, the total trend of mobile phone users’ distribution at midnight is reflected using a distribution scattered around the center of the city, while, during daytime, users gather around the city center.

**Figure 10:**
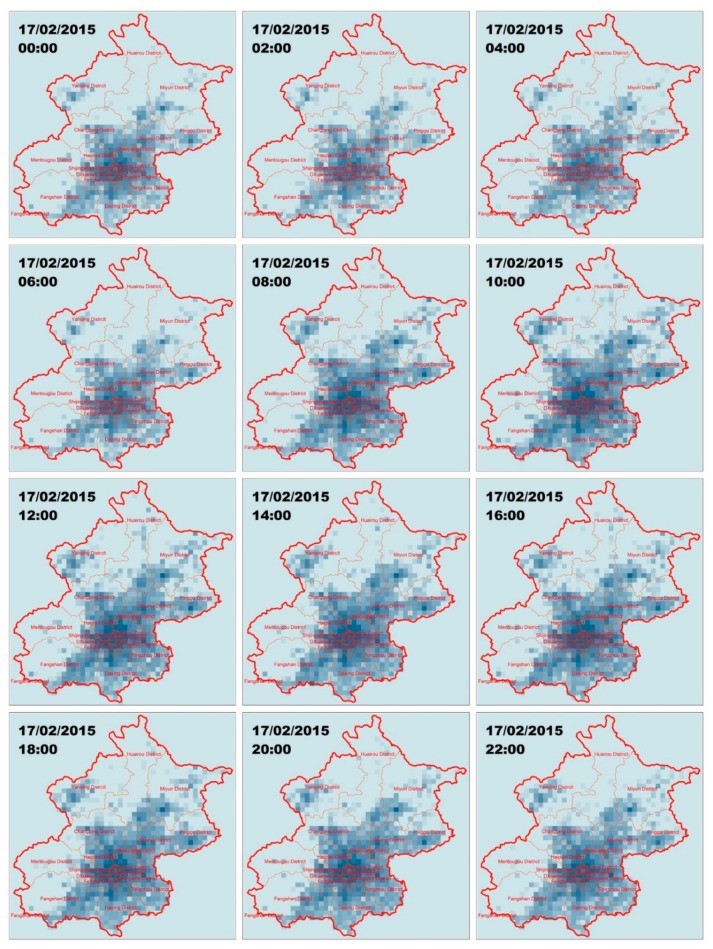
Mobile users’ distributions generated using kernel density estimation method on 17 February 2015 at a higher spatial resolution.

We extracted the corresponding mean density value into 2034 cells to represent the mobile phone distribution situation of every grid for a total of 192 times (the number of 2-h periods we examined for our study). The results of the decreased resolution had the same characteristics as the original graphs. Then, 180 matrices of 192 were used for training, while the other 12 matrices were used as the test data. The visualization of the last 12 matrices is shown in Figure 10.

### 5.2. Prediction Results for the ConvLSTM Model

**Figure 11:**
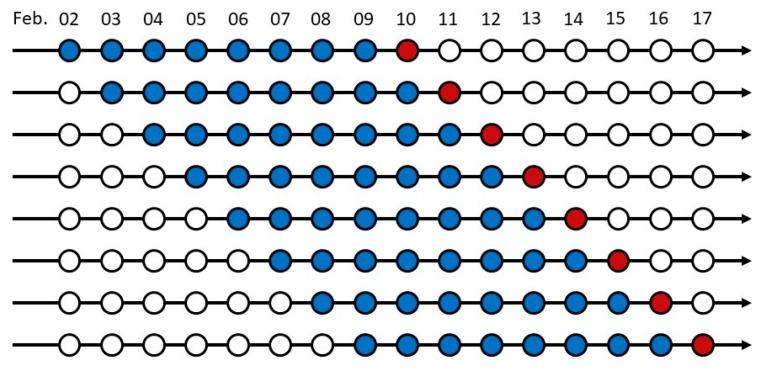
A cross-validation method for time-series data.

When testing the accuracy of ConvLSTM model, a cross-validation strategy is needed to assess the predictive performance of the models and to judge how they perform outside the training sample on a new dataset. There are many classical methods of cross-validation that can be used for machine learning and deep learning models, for example, K-fold cross-validation and leave-one-out cross-validation [69]. However, they cannot be used on time-series data because of the timing dependence when training and predicting [70]. To cater to the prediction of time sequences, an eight-split time-series cross-validation technique was used in our study, which is illustrated in Figure 11. The data were split into eight groups arranged in chronological order, with the blue circles indicating the test data (one circle presents one day, including a total 12 frames every two hours), the red circles representing the tested or predicted days (12 frames), and the hollow circles representing unused data. The number of blue circles in each split group was eight, whilst the number of red circles was one, which means we trained 96 frames to predict 12 frames using the ConvLSTM model eight times.

For each round of the prediction, the parameters of the model included the kernel size, which we proposed to be 3 × 3, with 40 convolutional filters that can extract important features from the convolution layers, with five units for each. In order to improve the generalization ability and to prevent over-fitting (which is the production of an analysis that corresponds too closely or exactly to a particular set of data, and may, therefore, fail to fit additional data or predict future observations reliably in machine learning or deep learning models), the recurrent weight dropout was set to 0.2 in the model; the number of training times (epochs) was set as 500, whilst the Adam optimizer [71] was used with a learning rate of 10^−3^ and a decay rate of 0.9.

**Figure 12:**
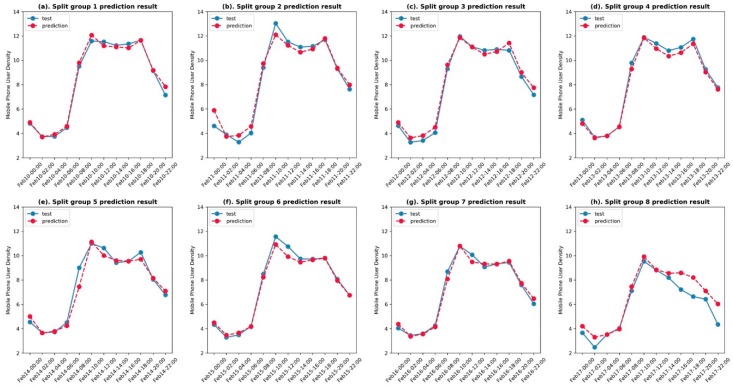
The relationship between the test data (blue circle) and prediction results (red circle) for the eight split groups (**a**–**h**), following the order from Figure 11, from top to bottom.

**Figure 13:**
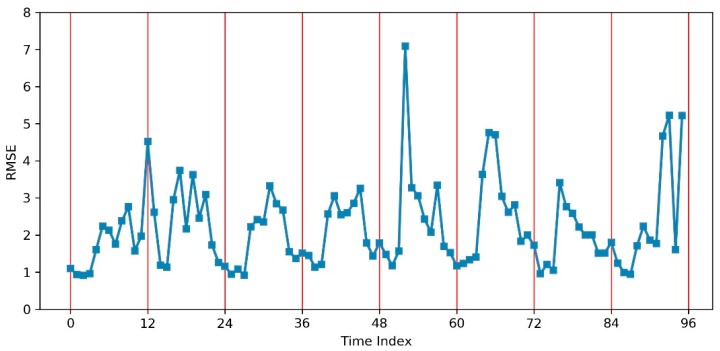
The root-mean-square error (RMSE) for every prediction result over a continuous time sequence.

**Figure 14:**
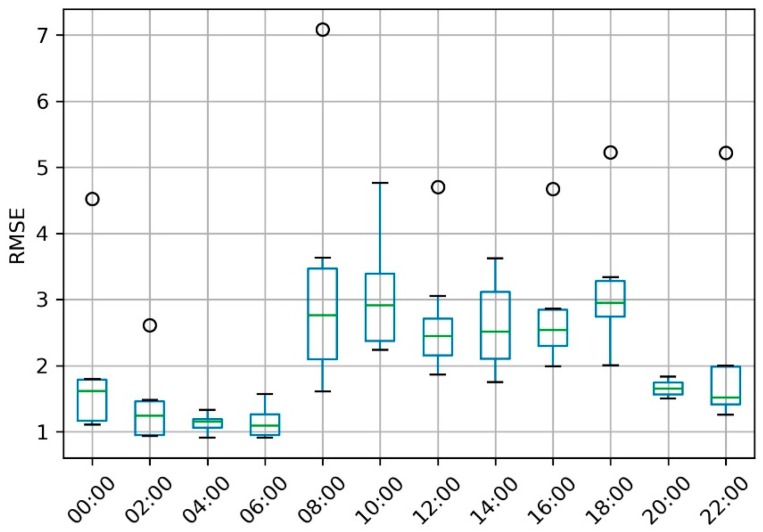
The results of eight split groups overlapping 12 time nodes in the predicted day.

Figure 12 shows the prediction result compared with the test data for eight cross-validation rounds. The accuracy of the predicted values using ConvLSTM appeared good in almost every round. Then, we calculated the root-mean-square error (RMSE) of every predicted and tested 48 × 48 frame. The RMSE results are plotted in Figure 13 over a continuous time sequence, showing that all eight rounds had an RMSE fluctuating between 0.5 and 7, while the values in round 5 were more unstable and had values above 7 (which were then treated as outliers), since the others were no larger than 6. Because we predicted 12 values in one whole day eight times, all RMSE results were extracted in the 12 time nodes from 12:00 a.m. to 10:00 p.m. to draw a boxplot, as shown in Figure 14. This box chart illustrates that, in the first four predicted time nodes, the accuracy reflected by the average RMSEs, which were lower than 2, was much higher, while the next six ones had values between 2 and 3. The RMSEs in the last two nodes were not higher than 2. However, the only outlier in this part was much higher than 5, which was caused by the prediction in round 8 (i.e., when the time node is equal to 96 in Figure 13).

**Figure 15:**
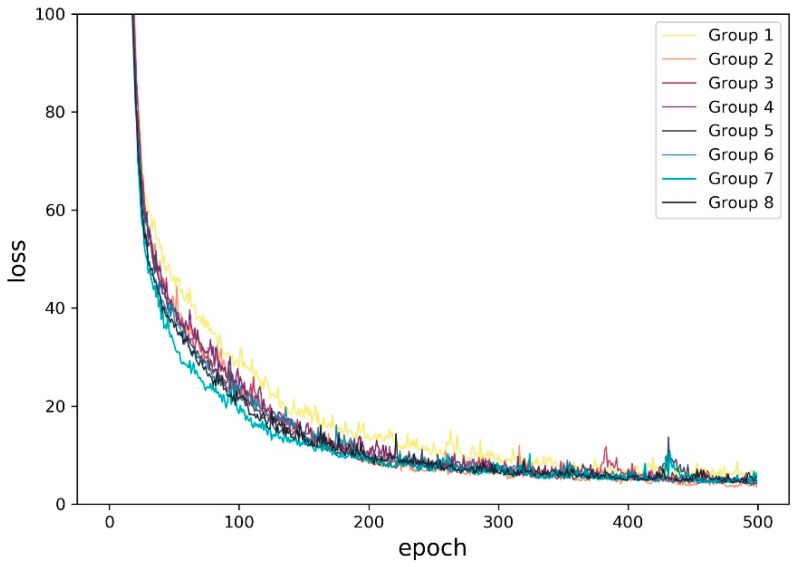
Predicted population density loss versus epoch number for the eight groups.

The relationship between the mean absolute error (MAE) and the training epoch of the results, which is called a loss function in machine learning or deep learning, is a non-linear function, as shown in Figure 15. It is illustrated that, when the epoch was greater than 200, the average value of the loss of the eight groups was stable around 5, and, when the epoch was 500, the average loss achieved the lowest value of about 4.4. A similar loss function was exhibited by the ConvLSTM model when we used it to predict the mobile phone user density distribution. In the next step, we also did this for the other baseline methods in order to compare them with the ConvLSTM model.

### 5.3. Prediction Results for the ConvLSTM Model versus the Two Baselines

**Figure 16:**
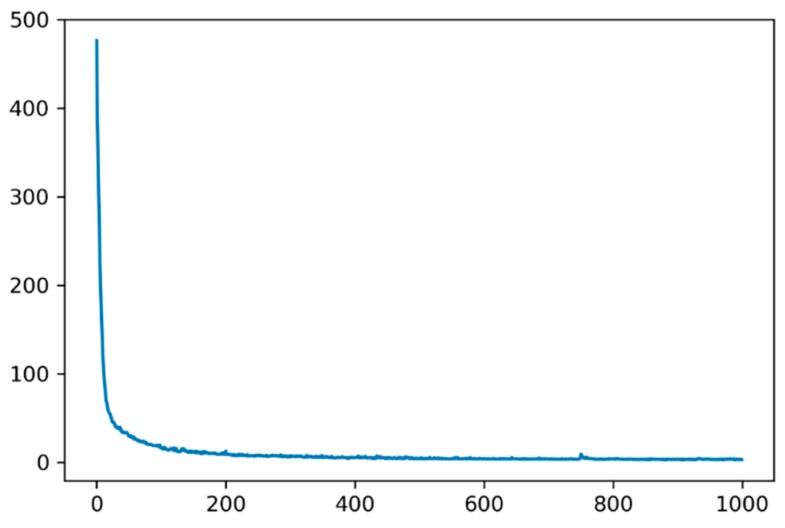
Predicted population density loss as a function of the epoch number or training time.

In order to maximize the potential of the ConvLSTM and baseline prediction models, when using ConvLSTM in this section, all data instances were 192 frames long, where 180 frames were used for the input and 12 frames were used for the prediction. The parameters of the model included the kernel size, which we proposed as 3 × 3, and the 40 convolutional filters, which can extract important features of the convolution layers in all three ConvLSTM layers with five units each. In order to improve the generalization ability and to prevent over-fitting of the model, the recurrent weight dropout was set to 0.2 in the model; the number of training times (epochs) was 1000, whilst the Adam optimizer was used with a learning rate of 10^−3^ and a decay rate of 0.9. The relationship between the loss of the results and the epoch was a non-linear function, as shown in Figure 16. It is illustrated that, when the epoch was greater than 200, the value of loss was stable around 4, and, when the epoch was 1000, loss achieved the lowest value of 3.23477. The assessment of the predicted results, both in time and space, is mentioned below.

#### 5.3.1. Results—Assessment of the Prediction Accuracy in Time

**Figure 17:**
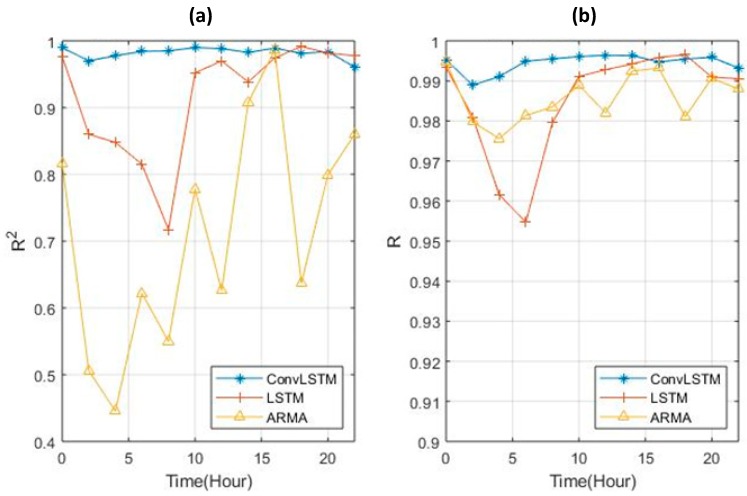
The change in *R^2^* coefficient (**a, left**) and *R* correlation coefficient (**b, right**) for the three methods.

The correlation analysis between the predicted and actual results was studied every two hours. It is obvious that, when using ConvLSTM to predict both the coefficient determination and correlation coefficient of the 12 2-h slots on 17 February, the values were much higher than the results predicted by ARMA and LSTM (0.99 as illustrated in Figure 17a,b). The result in Figure 17a starts at 0.99 at 12:00 a.m. and then decreases to the lowest value at time 2:00 a.m. (0.977). Then, it increases rapidly to 0.993 and keeps stable over the remaining period. The correlation coefficient has a very similar trend to the previous one, with its lowest value being 0.989. The values of both indices were not lower than 90%, which shows that the results of the prediction had a high accuracy in the temporal scale.

For the LSTM, the trends for the *R^2^* coefficient determination and the Pearson correlation coefficient (*R*) were similar to those of ConvLSTM, as these both decreased at the start and increased at the end. However, the fluctuation was much bigger for the former than the latter, which means that the prediction by LSTM was worse, and it was dependent on the size of the datasets. Furthermore, ARMA showed even more fluctuation compared to ConvLSTM, where it peaked at 4:00 p.m. and had its lowest point at 4:00 a.m. for the *R^2^* coefficient determination. Although, in Figure 17b, this got even higher than LSTM from 4:00 am to 8:00 am, ARMA still showed a lower accuracy than ConvLSTM.

It is worth mentioning that both *R^2^* and *R* in the ARMA and LSTM prediction processes fluctuated dramatically over the time. For the ARMA model, this was because it was a linear system model, which by default recognizes the input data as a Gaussian white noise sequence; hence, a relatively stationary sequence would be predicted by a linear system model with a sequence of white noise input, resulting in a larger error when the test value is fluctuating. In contrast, although LSTM was much more accurate than ARMA, even without convolution, a higher total error was generated from the accumulated error from each single cell in the grid. In contrast, because ConvLSTM takes the spatial autocorrelation into consideration, it created less accumulated error and the results of the correlation coefficient and coefficient determination were much better compared to the two baseline models.

**Figure 18:**
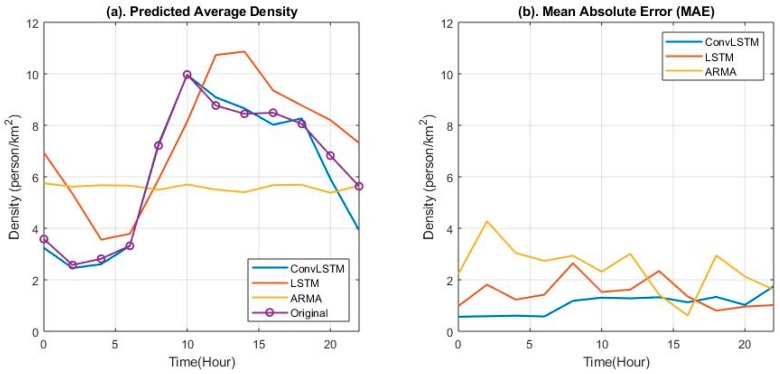
Transformation of the mean absolute error, and mean tested and predicted values for user density over all grids on 17 February 2015.

Figure 18a shows the transformation of the mean original and predicted values generated using the three methods over all grids during the whole test day, which can be used to intuitively explain the distance between the original and predicted values in the time series. Figure 18b shows the three mean absolute error (MAE) results generated by Figure 18a. The blue line representing ConvLSTM starts at 0.35, keeps stable to 6:00 a.m. and then increases slightly, and then remains stable to 8:00 p.m., before another increase at 10:00 p.m. The continuous rising trend of the MAE explains that the error of the predicted result processed by the ConvLSTM module grew with the accumulation of error. In contrast, both LSTM and ARMA showed bigger errors during the whole day except at 4:00 p.m. (yellow line) and after 6:00 p.m. (red line). ARMA results were much more stable when the density significantly changed, while LSTM showed a similar trend as time passed, albeit unsynchronized with the original line representing the test data.

#### 5.3.2. Results—Assessment of the Prediction Accuracy in Space

**Figure 19:**
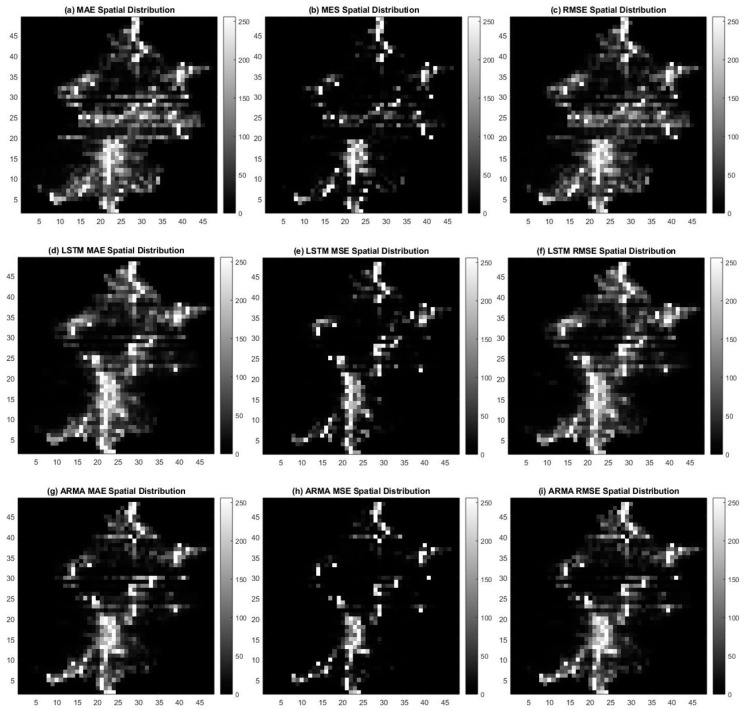
The spatial distribution of errors on 17 February 2015 for the three methods: (**a**–**c**) are the result maps for the mean absolute error (MAE), mean squared error (MSE), and root-mean-squared error (RMSE) distributions generated by ConvLSTM; (**d**–**f**) are the results generated by the LSTM model in the same order, while (**g**–**i**) are those generated by the ARMA model.

In terms of the spatial scale, we extracted the actual results with the corresponding original data and calculated the mean absolute error (MAE), mean squared error (MSE), and root-mean-squared error (RMSE) of every cell for the three approaches. Then, the spatial distribution of the three indices was visualized in the 48 × 48 matrix with 255 grayscale levels (Figure 19). The whiter regions in the graphs correspond to a higher error of prediction for user density. In contrast, the blacker regions mean the error is lower. Intuitively, the error distribution results from the corresponding user density in each cell represent the features in urban areas, which have more errors, while the sub-rural areas have fewer errors. Thus, the distribution of the error scan shows the effect of the prediction models in space.

Moran’s I analysis of the three indices is shown in Table 3, where the Moran’s indices of MAE for ConvLSTM, LSTM, and ARMA were 0.816785, 0.891316, and 0.859372, respectively. Moran’s indices of MSE were 0.733462, 0.742480, and 0.745777, and the equivalents of RMSE were 0.835418, 0.887711, and 0.858644. All Moran’s index values were between 0.7 to 1, which illustrates that the error of prediction had a positive spatial autocorrelation. However, the indices of ConvLSTM for MAE, MSE, and RMSE were lower than those for the other models. This means that the spatial relationship’s negative influence decreased for ConvLSTM, which considers spatial autocorrelation when using a convolution.

## 6. Conclusions

This study used a convolutional long short-term memory (ConvLSTM) module to predict the activity of mobile phone users’ distribution with a traditional time-series predicted model (ARMA) and a popular deep learning method (LSTM) as baselines for comparison. The evaluation results showed that the predicted density correlated much better with the original data at the temporal and spatial scales used when using ConvLSTM as compared to the other two methods, which do not consider the spatial autocorrelation. The MAE of the predicted results of ConvLSTM ranged from 0.6 to 1.8 over 17 February 2015, which means that the model was much more stable and accurate than the other two baseline methods. Moran’s I index for the error distribution was still lower than that of the other baseline methods in space, showing the positive effect on the errors caused by the spatial autocorrelation. Our proposed method, ConvLSTM, can help us better understand mobile phone users’ population dynamics and help more accurately calculate the population density for at least one day in advance based upon 15 days of history. Utilizing this method, population activity hotspots can be predicted, and this facilitates more dynamic and more efficient city resource and commercial deployment.

In the future, the method of converting mobile phone users into a population distribution and its application for a more dynamic prediction of people distribution using ConvLSTM will be investigated so as to improve the forecasting ability. In addition, we will use the mobile phone signal data and real people density data to analyze the demographics and people flow, so as to detect their residence and mobility paths, for which we can then use ConvLSTM to predict if their distribution can be converted into grids. Furthermore, some occupations, such as bus drivers and bank staff, based on their location and movement patterns, can be detected by mining their mobile phone data, which can then be used to predict the distribution of mobile users in a city via a spatial deep learning model.

## Figures and Tables

**Table 1:** Call detail record (CDR) data structure input for analysis. FID—feature identifier; ID—identifier.

FID	Name	Description
1	Time	Interactive time of users and base station
2	CI	Corresponding base station ID
3	Tmsi	Encrypted ID of users

**Table 2:** Base station index data structure.

FID	Name	Description
1	CI	Unique ID of base station
2	Lon, Lat	Latitude and longitude of base station location

**Table 3:** Moran’s I indices of the spatial distribution of the three methods. LSTM—long short-term memory; ConvLSTM—convolution LSTM; ARMA—autoregressive moving average; MAE—mean absolute error; MSE—mean squared error; RMSE—root-mean-squared error.

	MAE	MSE	RMSE
ConvLSTM	0.816785	0.733462	0.835418
LSTM	0.891316	0.742480	0.887711
ARMA	0.859372	0.745777	0.858644
